# Multi-parametric study of temperature and thermal damage of tumor exposed to high-frequency nanosecond-pulsed electric fields based on finite element simulation

**DOI:** 10.1007/s11517-016-1589-3

**Published:** 2016-11-16

**Authors:** Yan Mi, Shaoqin Rui, Chengxiang Li, Chenguo Yao, Jin Xu, Changhao Bian, Xuefeng Tang

**Affiliations:** 1 State Key Laboratory of Power Transmission Equipment & System Security and New Technology, School of Electrical Engineering, Chongqing University, No.174, Shazhengjie Street, Shapingba District, Chongqing, China; 2 The State Grid Tianjin Power Maintenance Company, No.42, Nankou Street, Hebei District, Tianjin, China

**Keywords:** Multi-parameters, Temperature, Thermal damage, Tumor, High-frequency nanosecond-pulsed electric fields, Finite element method

## Abstract

High-frequency nanosecond-pulsed electric fields were recently introduced for tumor or abnormal tissue ablation to solve some problems of conventional electroporation. However, it is necessary to study the thermal effects of high-field-intensity nanosecond pulses inside tissues. The multi-parametric analysis performed here is based on a finite element model of liver tissue with a tumor that has been punctured by a pair of needle electrodes. The pulse voltage used in this study ranges from 1 to 4 kV, the pulse width ranges from 50 to 500 ns, and the repetition frequency is between 100 kHz and 1 MHz. The total pulse length is 100 μs, and the pulse burst repetition frequency is 1 Hz. Blood flow and metabolic heat generation have also been considered. Results indicate that the maximum instantaneous temperature at 100 µs can reach 49 °C, with a maximum instantaneous temperature at 1 s of 40 °C, and will not cause thermal damage during single pulse bursts. By parameter fitting, we can obtain maximum instantaneous temperature at 100 µs and 1 s for any parameter values. However, higher temperatures will be achieved and may cause thermal damage when multiple pulse bursts are applied. These results provide theoretical basis of pulse parameter selection for future experimental researches.

## Introduction

Electroporation, as an intrinsically nonthermal phenomenon, is reversible when electric fields are used up to a specific level, but becomes irreversible at higher field levels. An irreversible electroporation (IRE) treatment includes electrode placement within the target region and delivery of a series of electric pulses of microsecond-scale single pulse duration with a low frequency. These microsecond-long high-voltage pulses can not only cause IRE on a cell membrane and then changes in the cell function, but can also induce biomedical effects such as apoptotic effects, anti-angiogenic effects and immune responses [35, 38]. Ultimately, IRE can achieve the goal of tumor ablation. IRE has recently also been considered as a nonthermal treatment modality to destroy tumors [14, 47, 55]. The most significant advantage of IRE is that it only affects the cell membrane while keeping the extracellular matrix (ECM) around the targeted cells intact by reducing Joule heating [47]. However, statistics from clinical trials show that muscle contraction appears during the pulsed electric field process and the patients suffer from muscle contraction discomfort during the treatment [7, 24]. When the width of the applied electric field pulse is reduced to the ns level, the electric field strength increases to the MV/m level, and the biological effects induced by nanosecond-pulsed electric fields (nsPEFs) are different from those of the aforementioned IRE. While no apparent irreversible electroporation phenomenon occurs on the cell membrane, a series of functional changes occur inside the cell, and then, apoptosis is induced [50, 51]. However, because of the high intensity of the pulsed electric fields that are applied to the electrodes, the treatment may cause surface discharges on the targeted tissue and skin burns.

To combine the advantages of both microsecond pulsed electric field (μsPEF) and nsPEF treatments, we introduced a high-frequency nsPEF protocol for treatment of tumors. Studies have shown that when a high-repetition-rate nsPEF is applied, the number of pulses makes a greater contribution to the killing effects than the field strength and the pulse width. In fact, an increase in the electric field pulse repetition frequency can inhibit patient muscle contraction [1, 10, 37, 39, 46, 57]. Therefore, we forecast that a high-repetition-frequency nsPEF increased to the 100 kHz level will effectively restrain patient muscle contraction. In addition, when the field strength is reduced to less than the breakdown field strength of air (10 kV/cm level), it will also effectively solve the problem of skin burns caused by the electrode discharge during nsPEF treatment. Consequently, the protocols that are proposed in this study can solve the problems of μsPEF and nsPEF treatments in cancer therapy, but also, through a synergistic effect, simultaneously perform the tasks and enhance the effects of inducing tumor cell necrosis and apoptosis. In addition, the high-frequency pulses can produce a more uniform electric field distribution to prevent tumor recurrence [2, 5]. Thus, this protocol is expected to provide a better outcome from cancer treatments.

Finally, it should be noted that high intensity pulsed electric fields will cause Joule heating, which should be avoided in electroporation applications, because temperature control is important even in IRE treatments. Lackovic et al. simulated the temperature distribution of a liver with needle electrodes during and after eight 100 μs, 1500 V/cm pulses and eight 50 ms, 250 V/cm pulses, with a repetition frequency of 1 Hz. The simulation results show that the Joule heating depends on the conductance of the tissue and the pulse parameters [32]. They also found that when the repetition rate increased from 1 Hz to 1 kHz, it could cause the tissue temperature to increase, but still by less than 3 °C [34]. Davalos et al. [15] elaborated on the determination of the temperature distribution and how to assess the thermal effects. They also investigated the temperature distribution and the thermal damage in the brain based on numerical models. The temperature was measured at the same time [21]. Thus, it is essential to pay greater attention to the thermal effects when tissue is exposed to high-frequency nsPEF treatment with a field strength that is greater than 1 kV/cm but less than 10 kV/cm. However, recent studies with regard to the temperature increase aspects of thermal damage are mainly concerned with the thermal effect under a given pulse parameter, or are simply research on the influence of a single parameter on tissue heating [12, 15, 21, 31, 32, 34, 41]. Therefore, in this study, we provide a multi-parameter analysis method to determine the relationship between the thermal effects and the pulse parameters (e.g., pulse width, pulse amplitude, repetition rate) and then to predict the temperature increase and the thermal damage. The results of this work can provide theoretical guidance for parameter selection in future tumor treatments using high-frequency nsPEFs.

## Methods

### Finite element model

**Fig. 1:**
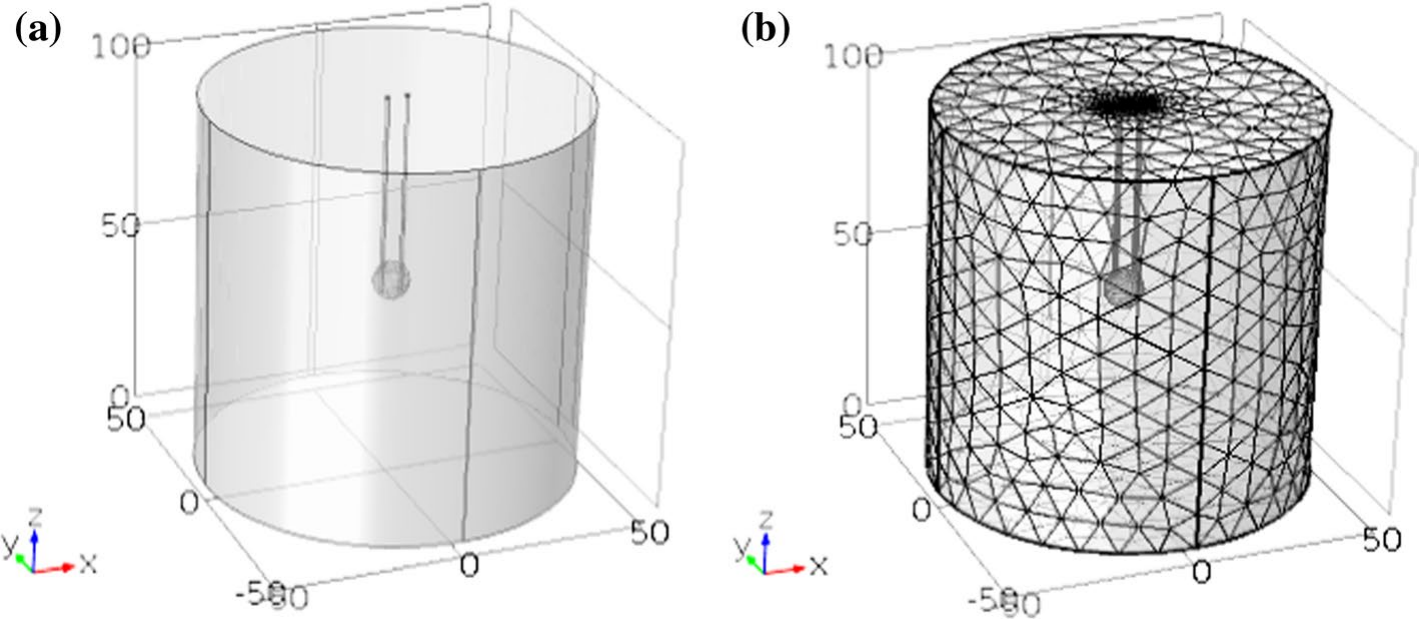
Geometrical model of tissue and electrodes. Liver diameter: 10 cm; liver height: 10 cm; tumor diameter: 1 cm; needle diameter: 1 mm; interelectrode distance: 5.4 mm; electrode insertion depth: 6 mm. **a** Geometrical model; **b** meshing model

This study was based on use of a finite element model by using finite simulation element analysis software of COMSOL Multiphysics to calculate the electrothermal coupling. The tumor model adopted a spherical geometry and the normal tissue around the tumor was represented by a cylinder, with its size as shown in Fig. 1. The liver diameter is 10 cm, and height is 10 cm. A pair of needle electrodes was used for hepatic tumor ablation. To maximize tumor ablation while reducing the damage to normal tissue around the tumor, the electrode needles were inserted directly into the tumor. The distance between the electrodes and the depth of penetration were all based on our previous optimization of a simulation for a tumor with a diameter of 1 cm [56]. The needle diameter is 1 mm, while the distance between electrodes is 5.4 mm and the insertion depth of electrodes is 6 mm. The purpose of the optimization was to make the best use of the electric fields and maximize the ratio of the tumor melted by the electric fields to the normal tissue ablation volume. According to Fig. 1b, the structure of free split tetrahedral was used. The smallest element size of electrodes and tumor is 0.4 mm, while the smallest element size of liver is 1.8 mm. The number of degrees of freedom is 405,643.

### Parameter model

**Fig. 2:**
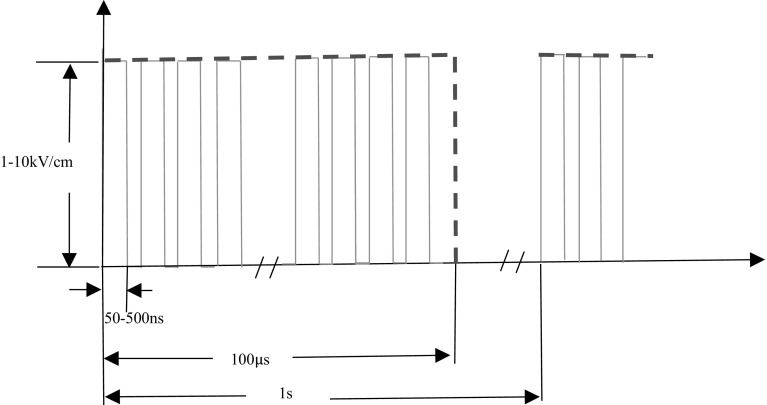
Schematic representation of pulse trains used in the simulations; pulse voltage: 1, 2, 3, 4 kV; pulse width: 50, 100, 250, 500 ns; pulse frequency: 100, 250, 500 kHz, 1 MHz; and repetition frequency of pulse bursts: 1 Hz

We introduced a type of high-frequency nanosecond pulses illustrated in Fig. 2. The electric field strength is from 1 to 10 kV/cm, the pulse width is range from 50 to 500 ns, while the repetition rate is from 100 kHz to 1 MHz. One of the characteristics of this pulse protocol is that the total pulse time is 100 µs no matter what the pulse width or repetition rate is. For example, when pulse width is 50 ns and pulse repetition rate is 1 MHz, the total high level duration is 5 µs. For pulse bursts with 1 Hz repetition frequencies, we run simulations for 1 s, which means one pulse burst.

The electrical properties and thermal properties of the tissue (rat liver) and the electrodes (stainless steel) were taken from the literature [3, 40, 52] and are listed in Table 1. The initial electrical conductivity of the rat liver that we used in this study was 0.067 S/m, and the conductivity of the tumor was 0.135 S/m [48]. It had previously been proved that tissue electrical conductivity increases because of electroporation during application of high-voltage pulses [52]. To analyze the temperature rise in the tissue, we ran simulations using the simplified model of the electrical conductivity. It was shown that when the tissue was electroporated, the electrical conductivity of the liver increased to 0.241 S/m and that of the tumor was 0.426 S/m [48]. The multiple of the increase in conductivity was in accord with the results of measurements by other researchers [40]. The reason for this simplification was that the parameters studied in this paper were already more enough. The purpose of the analysis was to study the relationship between the pulse parameters and the thermal effects. Therefore, it was most effective to simplify the calculation in this way. The threshold value of the field strength required to decide whether or not the tissues were electroporated was 800 V/cm [4, 14], which is considered to be the threshold for irreversible electroporation. The IRE pulse protocols are described as several 100 µs pulses with a frequency of 1 Hz. Because the total pulse time in this manuscript is 100 µs, it is reasonable to use 800 V/cm as the threshold.

**Table 1:** Material properties

	Mass density [*ρ* (kg/m^3^)]	Heat capacity [Cp (J kg^−1^ K^−1^)]	Thermal conductivity [*k* (W m^−1^ K^−1^)]	Electrical conductivity [*σ* (S/m)]	Blood perfusion [*ω*_b_ (1/s)]	Metabolic heat [*Q*_m_ (W/m^3^)]
Electrodes	70	1045	0.026	1e–5	–	–
Insulation	6450	840	18	1e8	–	–
Liver	1080	3540	0.52	0.067 (initial)	0.0005	4200
Tumor	1220	4180	0.6	0.135 (initial)	0.002	42,000

With regard to the blood perfusion and metabolic heat generation in biological heat transfer, the blood density and the heat capacity were *ρ*_b_ = 1000 kg/m^3^ and *c*_b_ = 4200 J/(kg K), respectively [28, 36]. Different values for blood perfusion and the metabolic heat of both the liver and the tumor are also listed in Table 1. The value of the blood perfusion and the metabolic heat of the tumor were both larger than that of the liver because of the specific characteristics of the tumor [16, 28, 36]. The temperature coefficient of electrical conductivity was 1.5%. Finally, the initial temperature and the arterial blood temperature were both 37 °C.

### Computing method

The electric potential distribution within the tissue was obtained by transient solution of the following:

1 $$- \nabla \cdot (\sigma \nabla \varphi ) = 0 ,$$

where φ is the electric potential and σ is the tissue conductivity. Heat transfer in the tissue can be modeled using the bioheat equation that was proposed by Pennes [43]:

2 $$\rho c\frac{\partial T}{\partial t} = \nabla \cdot (k\nabla T) + \rho_{\text{b}} w_{\text{b}} c_{\text{b}} (T - T_{\text{b}} ) + Q_{\text{m}} + Q$$

Here, *T* is the temperature, *t* is the time, *ρ*, *c* and *k* are the density, the heat capacity and the thermal conductivity of the tissue, *ω*_b_ is the blood perfusion, *ρ*_b_ and *c*_b_ are the density and the heat capacity of blood, *T*_*b*_ is the temperature of the arterial blood, *Q*_m_ is the metabolic heat, and *Q* is the Joule heating caused by the electric field.

3a $$E = - \nabla \varphi$$

3b $$J = \sigma E$$

3c $$Q = JE = \sigma |\nabla \varphi |^{2}$$

*E* is the electric field and *J* is the current density. The Pennes equation is a thermal–electric coupled field calculation, from which we can obtain the temperature of the biological tissue.

The electrical boundary condition at one electrode–tissue interface was set to be *φ* = *φ*(*t*), and *φ*(*t*) was the time-varying voltage. The other electrode–tissue interface was set at *φ* = 0. The remaining boundaries were treated as electrical insulation and are described by $\frac{{{\text{d}}\varphi }}{{{\text{d}}n}} = 0$. The outer surface of the liver tissue was set to be thermal insulation.

Thermal damage is a process that depends on both the temperature and the time, and occurs when the tissue temperature is elevated over an extended period of time. Some entries in the literature explain that damage can occur at temperatures as low as 42 °C if the exposure is long enough, while 73.4 °C is regarded as the target temperature for instantaneous thermal damage in liver tissue [8, 34, 42, 53, 54]. Also, some researchers believe that temperatures higher than 43–45 °C will lead to protein denaturation and destruction of the cell structure, which will eventually lead to cell necrosis. If the tissue temperature increases in a transient manner but to less than 45–50 °C, the effects may be negligible in terms of thermal injury [20, 33]. This is largely in line with the accepted viewpoint that if the temperature increase exceeds 8 °C, proteins will tend to denature [2]. Consequently, in this study we investigated the parameters that maintained temperature at a level below 44 °C. Simultaneously, the well-known Arrhenius first-order kinetic model was also used to evaluate the thermal damage to the tissue. The thermal damage *Ω* accumulated for time *t* is represented by the following equation [17]:

4 $$\varOmega (t) = A\int\limits_{0}^{t} {\exp ( - E/RT){\text{d}}t} ,$$

where *A* (1/s) is the pre-exponential factor, *E* (J/mol) is the activation energy, *R* (= 8.314 J/(mol K)) is the universal gas constant and *T (K)* is the absolute temperature. The damage process and the parameters are listed in Table 2. The parameters used in this computation are the pre-exponential factor *A* of 7.39e39 (1/*s*) and the activation energy *E* of 2.577e5 (J/mol), which represent protein coagulation [23].

**Table 2:** Parameters of damage process

Damage process	*E*(J/mol)	*A*(1/s)
Microvascular blood flow stasis	6.67e5	1.98e106
Cell death	5.064e5	2.984e80
Protein coagulation	2.577e5	7.39e39

5 $$P\,(\% ) = 100(1 - \exp ( - \varOmega ))$$

In terms of finite element modeling of the thermal damage, the value of *Ω* = 1 corresponds to a 63% probability of cell death, while the value of *Ω* = 4.6 represents a 99% probability of cell death due to the thermal effects. And the value of 0.53 is used as the threshold needed for thermal damage [21].

The computations use parameter scanning and transient solutions. Because the elapsed pulse time is very short (the total pulse length is 100 μs), particular attention was paid to the control of the time steps in the variable-step solver. We introduced time steps of 10 ns during the first 100 μs, and then extended the time step to 1 ms up to a total time of 1 s.

## Results

### Simulation results for temperature and thermal damage

**Fig. 3:**
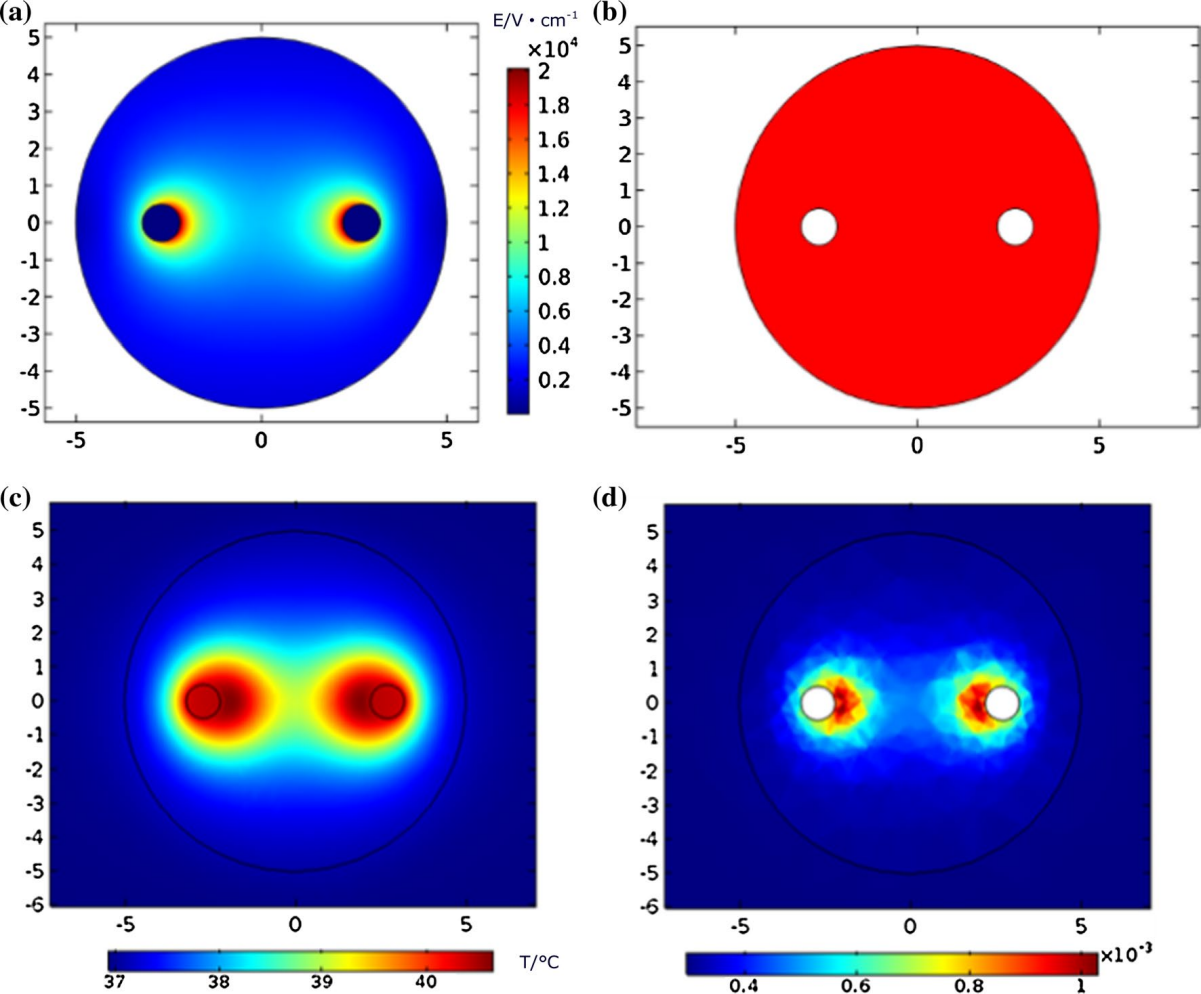
Spatial distribution (xy cross section, *z* = 50 mm) of electric field intensity (**a**), coverage of 800 V/cm (**b**), temperature (**c**) and thermal damage (**d**) in the tumor at the time point of 1 s when the applied voltage is 4000 V, the pulse width is 500 ns and the repetition rate is 1 MHz

When different pulsed voltages were applied, different temperature rises occurred in the tumor. The pulse voltage used in this analysis ranged from 1 to 4 kV, the pulse width ranged from 50 to 500 ns and the frequency ranged between 100 kHz and 1 MHz. The total pulse length was 100 μs, while the total simulation ran for 1 s. According to the simulation results, tumor electric field distribution when applying voltage of 4000 V is shown in Fig. 3a. Since the field of needle electrode is uneven, the electric field strength is higher on the interface of electrodes and tissue. And when applying pulse voltage of 4000 V, the coverage of 800 V/cm is illustrated in Fig. 3b. According to Fig. 3b, we can see that in that situation, the whole tumor was electroporated. In this way, we can not only obtain the temperature increase due to the pulsed electric fields, but we can also determine the maximum instantaneous temperature at 1 s related to the heat dissipation process of the tissue. The distributions of the maximum instantaneous temperature and the thermal damage at 1 s in the tumor are shown in Fig. 3c, d. The temperature indicates that the maximum instantaneous temperature at 1 s reaches 40.4 °C in the tumor near the electrodes. Additionally, the main area of temperature increase is focused around the tissue regions near and between the electrodes. The tissue in this region electroporated and the electrical conductivity consequently increased, as did the temperature. Figure 3d indicates that the thermal damage distribution is similar to that of the temperature, and the maximum thermal damage is only 0.0016 at the end of the 1 s simulation. At the same time, the shapes of the temperature and thermal damage distributions are the same as the results reported by other researchers [21].

**Fig. 4:**
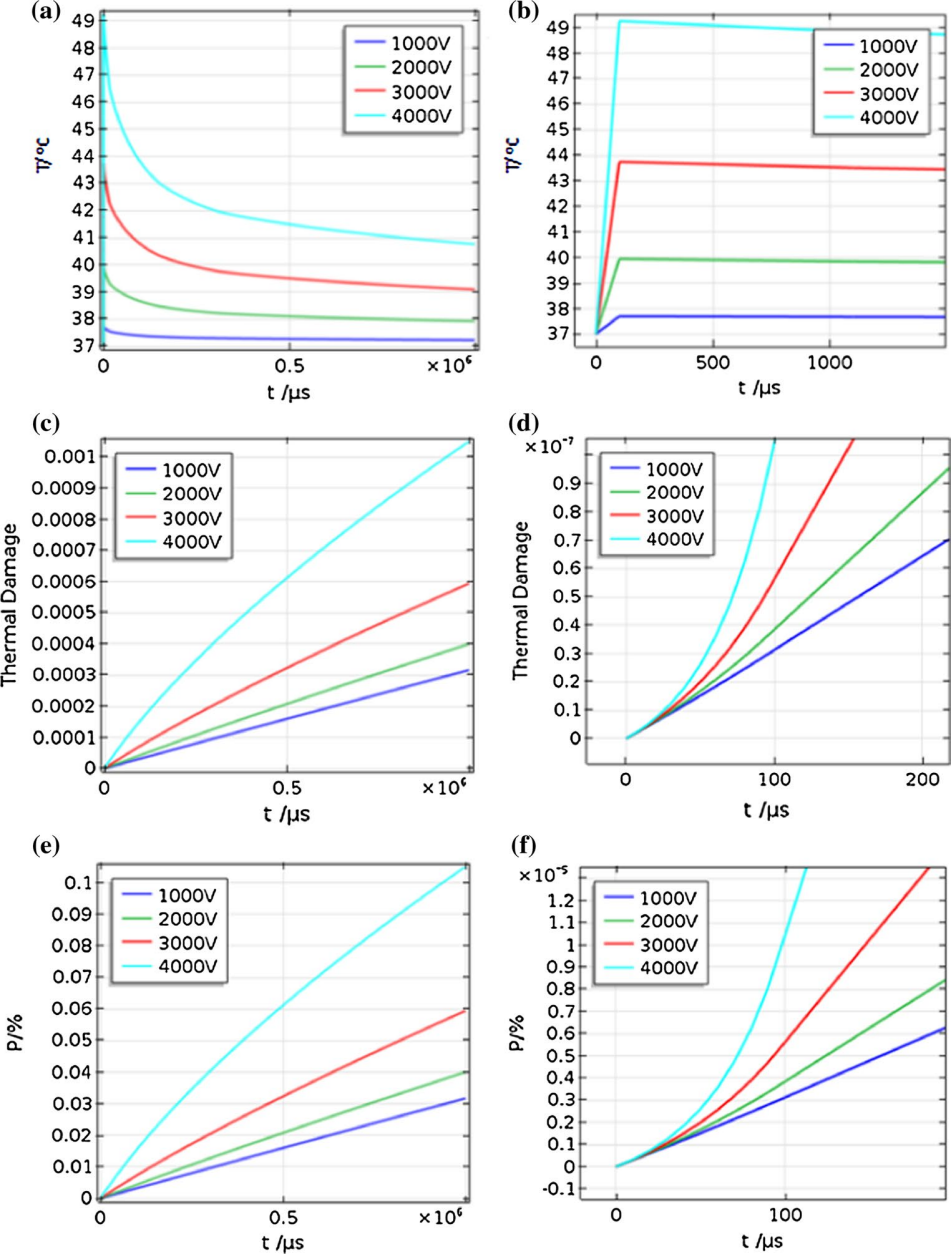
Changes in temperature, thermal damage and percentage of cell kill due to thermal damage with time: **a**, **c** and **e** show the temperature, thermal damage and percentage of cell kill due to thermal damage curves, respectively, when the pulse width is 500 ns and the repetition rate is 1 MHz; **b**, **d** and **f** show enlarged versions of (**a**), (**c**) and (**e**), respectively, focusing on the rise time. Different curves are the change under different pulse voltage: 1000, 2000, 3000 and 4000 V

Figure 4 demonstrates that the maximum instantaneous temperature at 100 µs increases approximately linearly with time during the pulses, and when the pulse is removed, the temperature decreases exponentially. From Fig. 4b, which shows a detailed view of Fig. 4a, we can see that the temperature actually increases stepwise over time. Figure 4c and d also shows that the maximum thermal damage changes nonlinearly with time within the first 100 μs, and then increases linearly. Figure 4e shows the change in percentage of cell kill due to thermal damage (*P*) with time, and Fig. 4f shows the enlargement of Fig. 4e. They were so much like the figure of thermal damage; just the value of *P* (%) is 100 times larger than thermal damage.

The maximum instantaneous temperature at 100 µs and maximum instantaneous thermal damage at 1 s that can be acquired are 49.26 °C and 0.0016, respectively.

### Relationship between thermal effects and pulse parameters

**Fig. 5:**
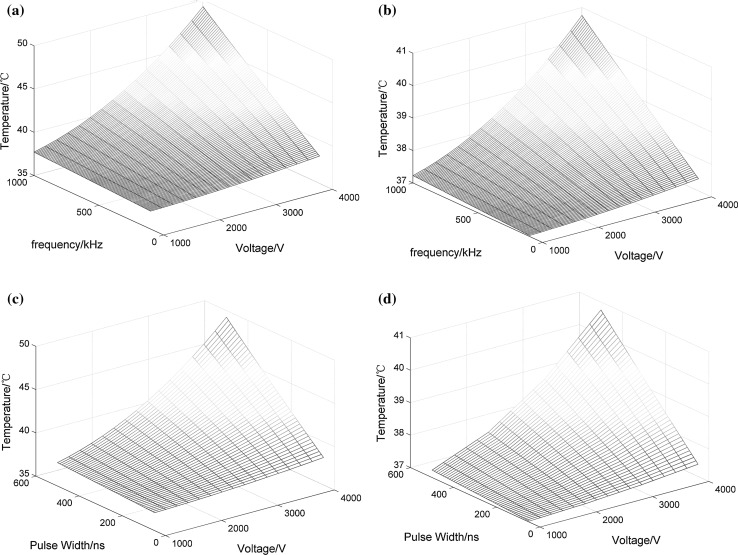
Relationship among tumor temperature, pulse voltage and frequency when the pulse width is 500 ns: **a** maximum instantaneous temperature at 100 µs and **b** maximum instantaneous temperature at 1 s; when the repetition frequency is 1 MHz, (**c**) and (**d**) show the relationships among tumor temperature, pulse voltage and pulse width

**Fig. 6:**
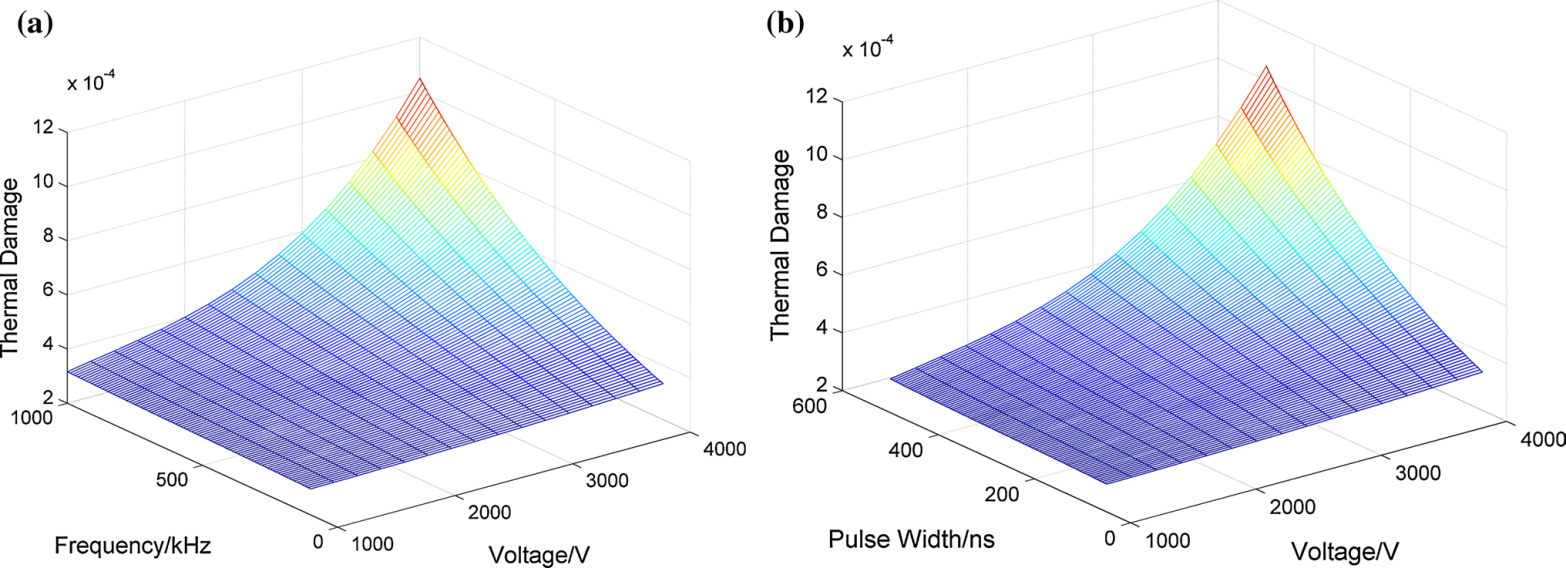
Relationship among thermal damage, pulse voltage, pulse width and frequency: **a** when the pulse width is 500 ns; **b** when the frequency is 1 MHz

The graphs are all drawn from the results of simulations by interpolation. Figure 5 displays the relationship between the temperature and the pulse parameters. More specifically, Fig. 5a, b shows diagrams of the maximum instantaneous temperature at 100 µs and the maximum instantaneous temperature at 1 s, respectively, when the pulse width is 500 ns. Figure 5c and d corresponds to a frequency of 1 MHz. From these figures, we can conclude that the relationship among the temperature, pulse width and frequency is linear, and the relationship between the temperature and the voltage follows a square law. Similarly, Fig. 6a illustrates the relationship among the maximum thermal damage in the tumor, the pulse voltage and the repetition frequency when the pulse width is 500 ns. The relationship among the thermal damage, the applied voltage and the pulse width is shown in Fig. 6b when the repetition frequency is 1 MHz.

### Determination of pulse parameters without causing thermal damage

**Fig. 7:**
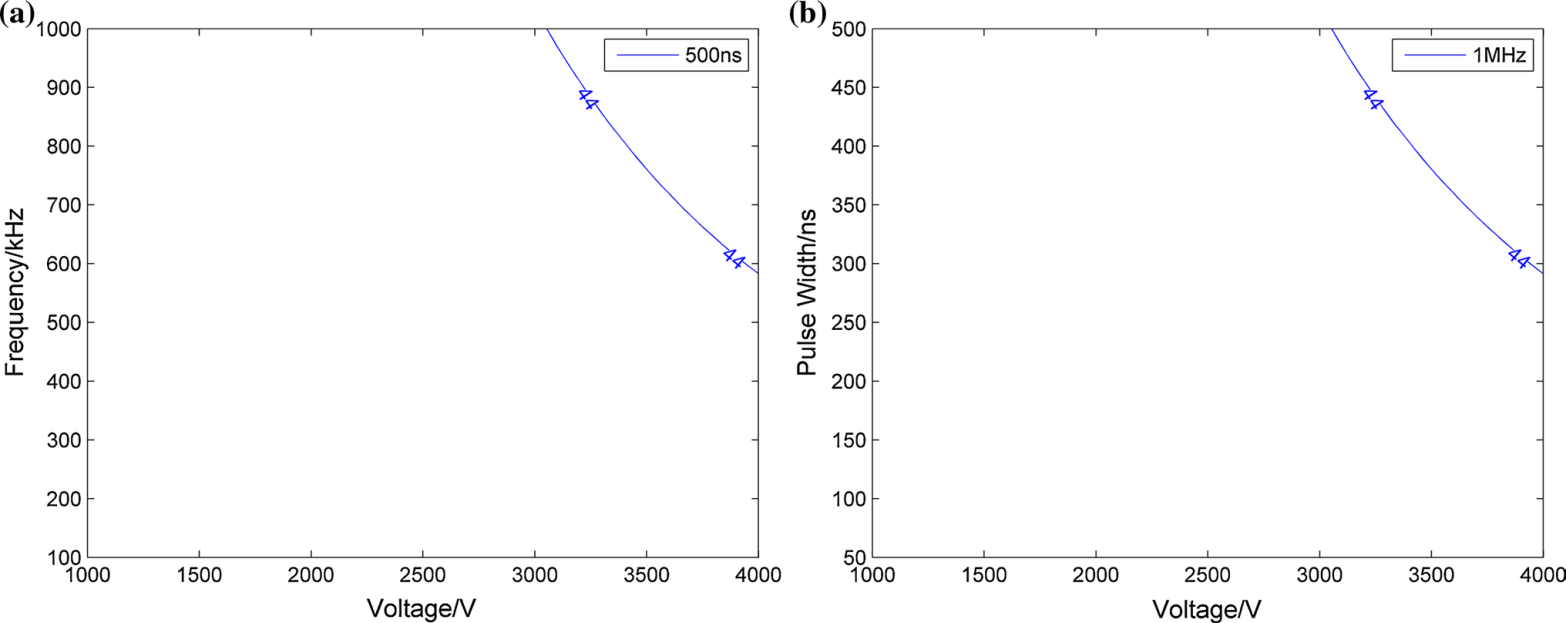
Temperature contours for 44 °C under different voltages, pulse widths and repetition frequencies: **a** when the pulse width is 500 ns; **b** when the frequency is 1 MHz

**Fig. 8:**
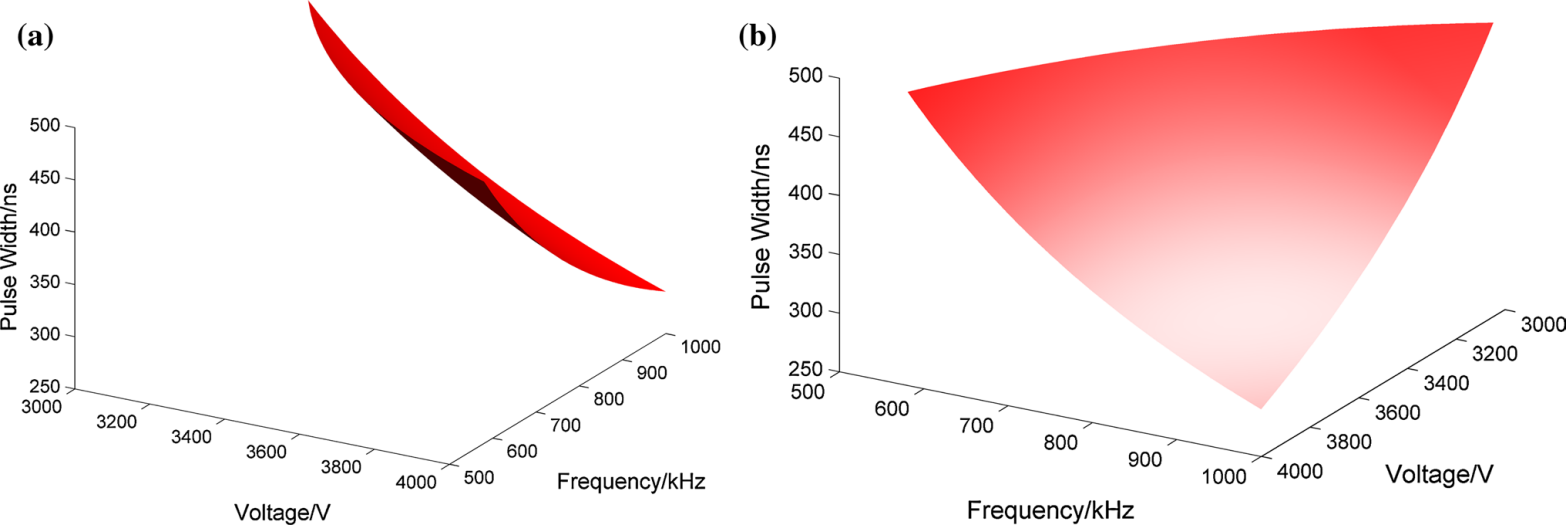
Curved surface for 44 °C under different voltages, pulse widths and repetition frequencies

Data were also processed to determine the upper specification limit to ensure that the temperature increase remains below 44 °C. The results are displayed in Figs. 7 and 8. Because our simulations only run for 1 s, the temperature does not get very high and the maximum instantaneous temperature at 100 µs can only reach 44 °C. Therefore, the following analyses were based entirely around this time point. In Fig. 7, via a two-dimensional parameter analysis, we can obtain the range of parameters that ensure that the temperature does not exceed 44 °C. When the value of the pulse width is a constant or when the frequency is constant, the parameters on the bottom left of the curve represent the desired parameter range. It is obvious that the temperature can only increase over 44 °C when the pulse width is 500 ns and the frequency is 1 MHz. For example, from Fig. 7a we can know that when pulse width is 500 ns and voltage is 4000 V, to make the temperature below 44 °C, repetition rate cannot be greater than 600 kHz. In the same way, when repetition rate is 1 MHz and voltage is 4000 V, pulse width cannot be greater than 300 ns. Also, we adopted a multiple parameter analysis method to evaluate the ranges of the three parameters for more specific results. Figure 8a, b shows the different angles of the three-dimensional curved surface, while the three axes represent the three pulse parameters: voltage, pulse width and frequency. From Fig. 8a, it is obviously a three-dimensional surface like a small piece of paper, and Fig. 8b is from the perspective of looking over the top right of Fig. 8a. The regions below the curved surface and close to the point of origin refer to temperatures of less than 44 °C, and the ranges of the three axes indicate which of the pulse parameters can be used at the same time. The results of our investigation, as shown in Figs. 7 and 8, can be used to provide theoretical guidance for parameter selection in practical experiments.

By calculating the electric field coupling with the thermal fields based on finite element simulations, the temperature and thermal damage profiles were obtained. On this basis, the data were analyzed to draw these figures and to pave the way for subsequent data fitting and estimation processes.

## Discussion

### Prediction of temperature and thermal damage under high-frequency nanosecond pulse bursts

This study provides an insight into the behavior of tissue thermal effects when short, square wave electric pulses of electroporation protocols are applied to the tissue. These protocols are different from the traditional IRE pulses and also from the pulse bursts of conventional nsPEF treatment. They are introduced as high-frequency nanosecond pulses, but the total pulse length is 100 μs. This kind of pulse can be considered as the use of microsecond pulses to modulate the nanosecond pulses to overcome the shortcomings of the two pulse protocols. However, it is noteworthy that these pulses may cause thermal damage to the tissue because of their high field strengths. This effect is important for planning of treatment protocols in the vicinity of sensitive structures such as blood vessels and nerves. Therefore, it is necessary to study the thermal effects under these high-frequency nanosecond-pulsed conditions. Based on the results from the simulations, we can obtain the maximum instantaneous temperature at 100 µs and maximum thermal damage at 1 s in the tumor under different voltages, pulse widths and repetition frequencies. The maximum instantaneous temperature at 100 µs and maximum thermal damage at 1 s that can be achieved are 49.26 °C and 0.0016, respectively, when the energy injection is a maximum. This suggests that thermal damage will not be caused within a single pulse burst. Because the relationships between the temperature and the pulse parameters were analyzed above, the maximum instantaneous temperature at 100 µs and the maximum instantaneous temperature at 1 s for the tumor can be fitted to the following formulas:

6 $$T_{\text{m}} \approx T_{0} + (1.5 \times 10^{ - 12} p_{\text{w}} fV^{2} )N,\;\left( {N\,{\text{is not too large}}} \right)$$

7 $$T_{\text{f}} \approx T_{0} + (4.8 \times 10^{ - 13} p_{\text{w}} fV^{2} )N,\;\left( {N\,{\text{is not too large}}} \right)$$

where *T*_m_ (°C) and *T*_f_ (°C) are the maximum instantaneous temperature at 100 µs and the maximum instantaneous temperature at 1 s in the tumor, respectively, *T*_0_ (= 37 °C) is the initial temperature, *p*_w_ (ns) is the pulse width, *f* (kHz) is the repetition frequency, *V* is the voltage applied to the electrodes and *N* is the number of pulse bursts. In this simulation, we run for only one pulse burst. However, it can be roughly estimated that the temperature will increase after multiple pulse bursts by a factor of *N*.

**Fig. 9:**
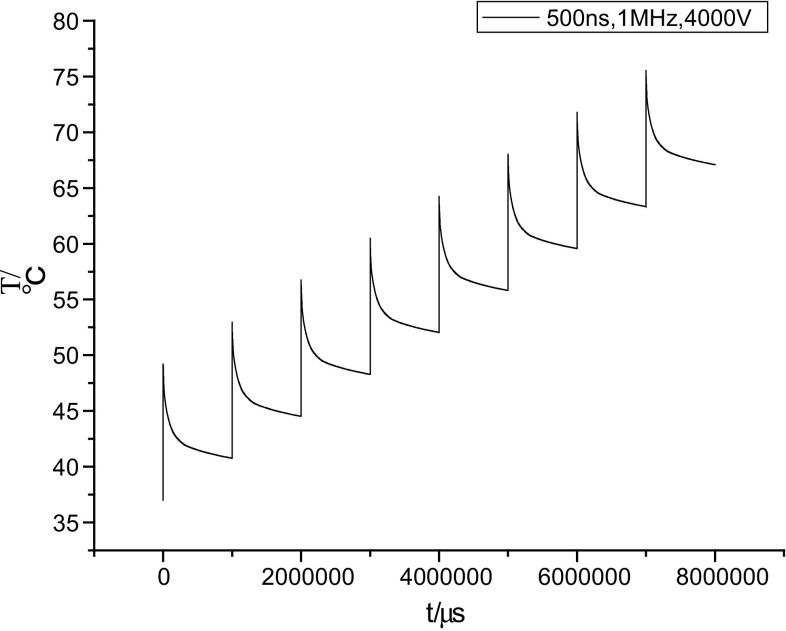
Temperature prediction curve

The temperature prediction curve is illustrated in Fig. 9, where the *x*-axis represents the number of pulse bursts and the *y*-axis represents the temperature increase in the tumor. The curve is when pulse voltage is 4000 V, pulse width is 500 ns, and pulse repetition is 1 MHz. From Fig. 9, we can see when increase the number of pulse burst to 8, temperature will reach to 75 °C, which may cause instantaneous thermal damage in tumor. According to this method, we can get the temperature rise under multiple pulse bursts. However, in fact, the temperature rise after each pulse burst is not the same as the number of pulses increases. When the temperature of biological tissue continues to rise, the cooling process is also become more obvious. If we assume that the temperature rise is same after each pulse burst, we can get an upper bound on the maximum temperature. Because the thermal damage is associated with the time integral of the temperature, it will reach such heights to cause the thermal damage when subjected to several bursts of pulses. This demonstrates the cumulative effect of the temperature and is related to the enclosed area below the curve. Consequently, we can roughly estimate the temperature increase in the tumor for a parameter choice that does not cause thermal damage. It should also be noted that when the treatment outcome is taken into consideration, we should impose more bursts of pulses. We should then wipe out a portion of the area near the electrodes because of the hot spots that always exist when performing an analysis of the thermal effects for parameter selection. However, it has still to be determined whether the removed segment is sufficient to meet the clinical requirements.

### Limitations of the simulations

Because this simulation aims to study the relationship between the thermal effects and the pulse parameters, we have drawn a number of conclusions from the results. However, there are also some limitations to our simulations:

1. First, this paper studies the influence of multiple parameters (voltage, pulse width and frequency) of high-frequency nanosecond pulses on the thermal effects. The number of values for each parameter that we discussed is only four. Even so, 64 parameter combinations are sufficient for a study of the rules, and more parameters could greatly increase the difficulty of the calculations. Unlike other studies in the literature, in which only a few parameters are studied [6, 19–24 31], this analysis was designed to be based on a multi-parameter perspective to determine the rules for fitting and estimating these parameters.
2. Second, some measurements have been performed to study the nonlinear increase in the tissue conductivity during IRE and nsPEF therapies when the tissues are exposed to sufficiently high electric fields [22, 26, 40, 45, 52]. However, few studies have been performed on the changing conductivity characteristics of tissue when subjected to high-frequency composite pulses. Bhonsle et al. [6] measured the conductivity before and after application of high-frequency bipolar pulses. The protocols in this simulation used high-frequency unipolar pulses and the changes in conductivity remain unclear. To simplify the calculations, we used a simple model of conductivity changes instead. The initial electrical conductivity of the rat liver that we used in this study was 0.067 S/m and the conductivity of the tumor was 0.135 S/m. When the tissue was electroporated, the electrical conductivity of the liver increased to 0.241 S/m and that of tumor increased to 0.426 S/m. Neal et al. used an equivalent circuit of a cell to analyze the bioimpedance behavior. A variable resistance was introduced to represent the macroscopic behavior of tissue under the influence of pulsed electric fields. When effective electric fields are applied, the resistance is a function of only the intra- and extracellular resistances because the variable resistance is short-circuited. The same effect is produced when the frequency of the pulses is high enough to make the capacitive component of the cell membrane short circuit [52]. The behavior of a single cell can be scaled to represent that of a larger tissue sample [18, 19]. In this way, we can obtain a method to estimate the increase in electrical conductivity that occurs when electroporation and the high-frequency components of the pulses produce a synergistic effect for further study.
3. Third, the threshold field for electroporation that was used in this study was 800 V/cm [4, 14]. However, as the pulse frequency increases, the permeabilization thresholds also increase [25, 49]. Different protocols may cause different increases in the threshold value. We can hardly set an increased electric field threshold for high-frequency nsPEF treatment casually without performing a great deal of preparatory experimental research. This article is intended to provide a simulation method to study the thermal effects, and therefore, it is acceptable to use the threshold field for irreversible electroporation.
4. Finally, this study of temperature and thermal damage has been performed on the basis of numerical simulations and thus lacks experimental verification. Despite this, the study is useful from the perspective of using multiple parameters to investigate the relationship between the thermal effect and the pulse parameters (voltage, pulse width and repetition frequency) under application of high-frequency nanosecond composite pulses.

### Future work

It is important to obtain accurate values of the changes in conductivity to calculate the electric field distribution and predict the outcomes of use of high-frequency pulses and the thermal effects. There are many studies that have measured the increases in tissue conductivity during electroporation-based protocols [9, 11, 26, 27, 29, 45]. The feasibility of using electrical impedance tomography [11, 13] and magnetic resonance electrical impedance tomography [29, 30] to monitor the electric field distributions has also been suggested. Our next work is to measure the conductivity of tissue when subjected to high-frequency nanosecond pulses, and to verify the effects of the high-frequency components on the electrical conductivity.

Temperature measurement is also vital to verify the accuracy of the models by comparing the experiment results with those of the theoretical calculations. Garcia et al. used a fiber optic temperature sensor to measure the temperature inside the tissue [12, 22]. A thermocouple was used by Pliquett et al [44]. for bulk temperature measurements, while temperature-sensitive liquid crystal was also used to measure the surface temperature. A thermal camera can also be used to capture the surface temperatures [6].

From a local viewpoint, the protocol proposed in this study can be viewed as use of high-frequency nanosecond pulses, but it also has the characteristics of microsecond pulses overall. Further research is necessary to assess the treatment outcomes, including the mechanism when a tumor is exposed to such pulses. In general, when applying IER pulses, it will appear on the cell membrane of several nanometers to several tens of nanometers pores. The poles with several nanometers size will recover while pores with tens of nanometers size will continue to expand to several hundred nanometers or micrometers, which are irreversible. But when applying low-frequency nsPEF, it will also appear on pores of several nanometers size, which are reversible. So when apply high-frequency nsPEF, it will produce some small nanopores at the beginning, and then, because the total pulse time is 100 μs, the nanopores may be expanded like IRE. We assume that nsPEFs can produce nanopores on the cell membrane, which will promote irreversible electroporation on the cell membrane by μsPEF. When the outer membranes have been corrupted, this will have beneficial effects for electroporation of the organelle membrane to induce apoptosis. There is a hypothesis that nsPEFs combined with μsPEFs are applied on both the inner and outer membrane, inducing tumor cell necrosis and apoptosis by a direct killing effect and slow indirect regulation, but numerous experiments are still required to verify this hypothesis.

## Conclusions

In this study, we have presented a type of pulse protocol for electroporation-based therapies. The pulse voltage used is in the range from 1 to 4 kV, and the pulse width ranges from 50 ns to 500 ns, while the repetition frequency is in the range between 100 kHz and 1 MHz. The total pulse length is 100 μs, and the repetition rate of the pulse bursts is 1 Hz. To analyze the thermal effect on the tumor, simulation models were developed based on finite element methods. Results from the simulations indicate that the maximum instantaneous temperature at 100 µs is up to 49.26 °C, and the maximum instantaneous temperature at 1 s and maximum instantaneous thermal damage at 1 s reach values of 40.4 °C and 0.0016, respectively, during a single pulse burst. Through multi-parameter analysis, we can obtain rules on how the pulse parameters affect the temperature and the thermal damage. By parameter fitting, maximum instantaneous temperature at 100 µs and 1 s for any parameter value after a single pulse burst or multiple pulse bursts can be calculated. In addition, higher temperatures are likely to be achieved and may cause thermal damage, based on parameter estimation when several bursts of pulses are applied. The results of temperature and thermal damage calculations performed using different high-frequency nsPEF parameters can provide a theoretical basis for selection of parameter options for experimental research.
